# Prediction of transition from ultra-high risk to first-episode psychosis using a probabilistic model combining history, clinical assessment and fatty-acid biomarkers

**DOI:** 10.1038/tp.2016.170

**Published:** 2016-09-20

**Authors:** S R Clark, B T Baune, K O Schubert, S Lavoie, S Smesny, S M Rice, M R Schäfer, F Benninger, M Feucht, C M Klier, P D McGorry, G P Amminger

**Affiliations:** 1 Discipline of Psychiatry, Royal Adelaide Hospital, University of Adelaide, Adelaide, SA, Australia; 2 Orygen, The National Centre of Excellence in Youth Mental Health and Centre for Youth Mental Health, The University of Melbourne, Melbourne, VIC, Australia; 3 Department of Psychiatry, University Hospital Jena, Jena, Germany; 4 Department of Child and Adolescent Psychiatry, Medical University of Vienna, Vienna, Austria; 5 Department of Pediatrics and Adolescent Medicine, Medical University of Vienna, Vienna, Austria

## Abstract

Current criteria identifying patients with ultra-high risk of psychosis (UHR) have low specificity, and less than one-third of UHR cases experience transition to psychosis within 3 years of initial assessment. We explored whether a Bayesian probabilistic multimodal model, combining baseline historical and clinical risk factors with biomarkers (oxidative stress, cell membrane fatty acids, resting quantitative electroencephalography (qEEG)), could improve this specificity. We analyzed data of a UHR cohort (*n*=40) with a 1-year transition rate of 28%. Positive and negative likelihood ratios were calculated for predictor variables with statistically significant receiver operating characteristic curves (ROCs), which excluded oxidative stress markers and qEEG parameters as significant predictors of transition. We clustered significant variables into historical (history of drug use), clinical (Positive and Negative Symptoms Scale positive, negative and general scores and Global Assessment of Function) and biomarker (total omega-3, nervonic acid) groups, and calculated the post-test probability of transition for each group and for group combinations using the odds ratio form of Bayes' rule. Combination of the three variable groups vastly improved the specificity of prediction (area under ROC=0.919, sensitivity=72.73%, specificity=96.43%). In this sample, our model identified over 70% of UHR patients who transitioned within 1 year, compared with 28% identified by standard UHR criteria. The model classified 77% of cases as very high or low risk (*P*>0.9, <0.1) based on history and clinical assessment, suggesting that a staged approach could be most efficient, reserving fatty-acid markers for 23% of cases remaining at intermediate probability following bedside interview.

## Introduction

The concept of clinical ultrahigh risk (UHR) for psychosis has been developed to facilitate early detection and intervention and is defined by a cluster of subthreshold psychotic symptoms affecting perception (for example, hallucinations) and thinking (for example, ideas of reference, odd beliefs or magical thinking) or trait risk factors (for example, family history of psychosis), accompanied by impairment in day-to-day function.^1^ Recent meta-analysis shows that less than 30% of UHR patients will have transitioned to psychosis 3 years after identification.^2^ Such poor specificity of UHR criteria poses a major challenge to indicated prevention for at-risk patients.^3^

Previous work has identified clinical and biological predictors for transition from UHR to psychosis. Static clinical features such as age, gender, duration of symptoms, traumatic experiences, a history of substance use and impaired premorbid psychosocial functioning are all associated with an increased risk of transition.^4, 5, 6, 7^ Dynamic clinical factors include the extent of baseline mood and psychotic symptoms.^1, 8^ In addition, specific patterns of cognitive function, particularly deficits in verbal fluency, memory and emotional processing,^9, 10^ as well as impaired general function are associated with a higher risk of transition.^6, 11^ Biological predictors include abnormalities of structural and functional neuroimaging,^12^ electrophysiology,^13, 14, 15^ and genetic^16, 17^ and proteomic markers.^18^ Individually all of these predictors are of small effect size and integrative models are required to combine multimodal information in the clinical setting to inform the risk of transition for an individual patient.^3^ Beyond simple multivariate regression, machine-learning techniques, including support vector machines, linear discriminant analysis and k-nearest neighbor analysis, have been utilized to extract patterns across multiple variables in large data sets. These approaches have been particularly useful for the analysis of neuroimaging data sets containing many thousands of variables.^19^ Recently, Amminger *et al.*^20^ used a supervised machine-learning technique known as Gaussian Process Classification to identify the pattern of fatty acids derived from erythrocyte membrane associated with 12-month functional outcomes in a UHR sample. Few studies have extended these techniques to include multimodal data.

We have recently reported on a Bayesian modeling technique that may have utility in overcoming this problem.^3^ The odds ratio form of Bayes Rule^21, 22^ offers a relatively simple method to combine multivariate data in a probabilistic manner that approximates the stepwise accumulation of data collected in the diagnostic process.^3, 23, 24, 25, 26, 27^ Using this type of modeling, we demonstrated that predictive accuracy at first presentation with UHR could be improved by combining several modes of assessment (for example, cognitive, neuroimaging and electrophysiology). Our simulation based on published results suggests that even in a help-seeking cohort with a high probability of transition to a first episode of psychosis (FEP) at least two other modalities of assessment in addition to UHR criteria are required at initial presentation to differentiate patients into high-, intermediate- and low-risk groups.^3, 27^ In the present study, we sought to extend and validate our simulation model, using an original single data set containing blood-based and resting quantitative electroencephalogram (qEEG) biomarkers and clinical data from a sample of UHR patients followed up for 1 year.^28^

We considered erythrocyte membrane fatty acids and markers of oxidative stress as a reflection of the balance of protective and degenerative mechanisms acting on neuronal membranes in psychotic illness.^29^ Oxidative stress occurs when reactive oxygen or nitrogen molecules generated through biochemical reactions interact with membrane lipids, proteins and DNA causing oxidative damage.^30, 31, 32^ In FEP patients, antioxidant enzymes and cofactors such as superoxide dismutase and glutathione (GSH) are low, whereas oxidative metabolites of membrane phospholipids such as thiobarbituric acid-reactive substances and malondialdehyde are increased, indicating higher rates of membrane damage.^30^ In patients with established schizophrenia, markers of oxidative stress are related to the severity of psychotic symptoms, impairment in cognitive and general function and brain volume loss.^29, 30, 33, 34^

Polyunsaturated fatty acids (PUFAs) are themselves vulnerable to oxidation but have anti-inflammatory and neuroprotective properties.^31, 35^ Omega-3 fatty-acid supplementation acts to restore neuronal membrane bilayer composition resulting in normalized membrane fluidity and function. Supplementation also inhibits phospholipase A2 activity, reducing the subsequent production of arachidonic acid, inflammatory eicosanoids and cytokines. The metabolism of omega-3 PUFAs by cyclo-oxygenase and lipo-oxgenase also produces anti-inflammatory resolvin and protectin mediators.^35, 36^ Omega-3 supplementation reduces the rate of transition to FEP for up to 7 years in UHR.^28, 37^ High PUFA levels in schizophrenia are associated with improved myelin integrity, reduced psychotic symptoms and better function.^20, 38, 39, 40^ However, low PUFA levels are associated with psychotic illness.^41, 42, 43^ Specifically, low nervonic acid, a monounsaturated very-long-chain fatty acid involved in myelin synthesis, is associated with transition to FEP.^40^

We chose qEEG as a further mode for psychosis risk modeling to reflect underlying abnormalities in oscillation synchrony of neuronal circuits associated with symptoms in psychotic illness.^44^ Increased slow wave (delta and theta) activity in frontal regions is common in psychosis^45, 46^ and is associated with negative symptoms in UHR, FEP and established schizophrenia.^15, 47, 48^ One recent study found that increased frontal delta and theta spectra and decreased alpha peak frequency significantly predicted transition to FEP.^49^

In the present study, we used the odds ratio form of Bayes' Rule to develop a multimodal model combining historical and clinical data and a set of biomarkers identified from oxidative, fatty-acid and qEEG candidates. We hypothesized that combined multimodal data are better than single markers in predicting transition, and that biomarkers of oxidative stress, PUFA levels and qEEG could be relevant for this prediction. By adding these biomarkers we sought to improve the specificity of current UHR criteria to reduce the false-positive rate, allowing more definitive indicated prevention.

## Materials and methods

### Study participants

We analyzed data from the placebo group (*n*=40) of a 12-week trial of the effect of omega-3 PUFA supplementation on 12-month transition to psychosis in a help-seeking UHR cohort. Following Morrison *et al.,*^50^ UHR was identified with the Positive and Negative Syndrome Scale (PANSS) using criteria proposed by Yung *et al.*^51^ Inclusion criteria included a history of attenuated positive psychotic symptoms, transient psychosis or trait plus state risk factors as indicated by family history in a first-degree relative of psychosis plus a decrease in functioning of 30% in the Global assessment of function (GAF) scale.^28, 50, 51^ These criteria were implemented before the availability of tools such as the Comprehensive Assessment of At-Risk Mental States (CAARMS) but are equivalent. Findings from this trial have been included in meta-analyses with studies using the CAARMS.^52, 53, 54^ The Structured Clinical Interview for DSM-IV-TR Axis I Disorders was used to confirm psychiatric diagnoses at baseline and 12-month follow-up in the original study. Exclusion criteria included the following: history of a previous psychotic disorder or manic episode (both treated or untreated); substance-induced psychotic disorder; acute suicidal or aggressive behavior; a current DSM-IV diagnosis of substance dependence (except cannabis dependence); neurological disorders (for example, epilepsy); intelligence quotient of less than 70; structural brain changes apparent on magnetic resonance imaging, except for enlargement of the ventricles or sulci or other minor abnormalities without pathological relevance (for example, white matter lucencies or temporal horn asymmetry); previous treatment with an antipsychotic or mood-stabilizing agent (1 week); having taken omega-3 supplements within 8 weeks of being included in the trial; laboratory values more than 10% outside the normal range for transaminases, thyroid hormones, C-reactive protein or bleeding parameters; and another severe intercurrent illness that may have put the person at risk or influenced the results of the trial or affected their ability to take part in the trial. The study was approved by the Medical University of Vienna Ethics Committee.

Two hundred and fifty-six individuals consecutively presenting to the psychosis detection unit of the Department of Child and Adolescent Psychiatry at Vienna General Hospital between April 2004 and May 2006 were assessed for eligibility, 81 of whom met the inclusion criteria and consented to the study. The details of this study have been described elsewhere.^28^

### Experimental design

In the clincal trial a computer-generated random sequence based on a block-randomized design (two strata with block size of four within each stratum) was used. Random assignment to omega-3 treatment or placebo group was stratified using the Montgomery Asberg Depression Rating Scale (total score <21 or ⩾21), as depressive symptoms may affect illness progression. The primary outcome measure of the trial was transition to psychosis defined using the Positive and Negative Symptoms Scale (PANSS; score of 4 on hallucinations, 4 on delusions or 5 on conceptual disorganization, sustained for at least 1 week). Data available included historical risks (gender, age, family history of psychosis, duration of symptoms at presentation and any history of drug use), standardized clinical assessments (PANSS-positive, -negative and general scales, Montgomery Asberg Depression Rating Scale and GAF score) and blood biomarkers including measures of oxidative stress, fatty acids and resting brain activity. The details of laboratory analyses of oxidative stress markers, fatty acids and of electrophysiological recordings are described elsewhere.^15, 35, 55, 56^ Total fatty acids and nervonic acid were analyzed from erythrocyte membranes. The fatty-acid-releasing enzyme phospholipase A2 was analyzed in serum. Oxidative markers included the enzyme superoxide dismutase, GSH (reduced form), oxidized GSH (GSSG)) and the ratio of reduced to oxidized GSH (GSH/GSSG) were measured in erythrocyte lysates.^56^ Resting EEG recordings, log-transformed absolute power, were obtained at each of 19 electrodes arranged in the 20/10 configuration for delta (1.0–4.0 Hz), theta (4.0–8.0 Hz), alpha (8.0–12.5), beta1 (12.5–18.5 Hz) and beta2 (18.5–30.0 Hz) frequency bands. On the basis of analysis by Lavoie *et al.,*^15^ delta EEG power was averaged between all electrodes in the whole frontal, the frontal left (F3, F7 and FP1) and the frontal right (F4, F8 and FP2) regions. Similarly, alpha power was averaged in the same frontal regions and beta1 power was averaged in the parieto-occipital, parieto-occipital left (P3 and O1) and parieto-occipital right (P4 and O2) regions. We included delta frontal, alpha frontal and beta1 occipitoparietal values as predictors for receiver operating characteristic curve (ROC) analysis because of their association with psychotic symptoms in Lavoie *et al.*^15^

### Statistical analysis

All statistical analyses were performed using the Medcalc statistical software package.^57^ The power of this analysis was 0.8 at a moderate effect size of 0.44 based on fixed model regression (F=2.31). Odds ratio form of Bayes' Rule models and probability plots were constructed in Microsoft Excel 2011 for Mac (Version 14.4.5). As a first step, we calculated ROCs^58^ for all predictor variables using the Youden index to establish the optimal threshold for cutoffs of continuous variables.^59^ We selected variables with area under the ROC (AUROC) significantly greater than 0.5 (*P>*0.05) for the final odds ratio form of the Bayes' Rule model. At an AUROC of 0.5, a test has no ability to differentiate between two groups.^60^ To internally validate the full model, calculations of 95% confidence intervals for model sensitivity at fixed specificity were bootstrapped at 1000 iterations with the Medcalc program using the BC_a_ algorithm.^61, 62^ Oxidative markers and qEEG parameters were not significantly associated with transition and were excluded from further modeling.

As a second step, we calculated positive and negative likelihood ratios (LRs) for each significant baseline predictor using the following formulae:^26^ Positive Likelihood ratio (LR+)=Sensitivity/(1−Specificity); Negative Likelihood ratio (LR−)=(1−Sensitivity)/Specificity. The LR+ is a measure of the probability of a positive test result in affected persons divided by the probability of a positive result in non-affected persons. In contrast, the LR− is a measure of the probability of a negative test result in diseased persons divided by the probability of a negative result in non-diseased persons.^26^ A test with a LR equal to 1 has no predictive value. When the LR+ is greater than 1, a positive test result suggests an increased probability of the disease. A LR+ of 10 or greater provides strong evidence to rule in a diagnosis. When the LR− is less than 1, a negative test result indicates a reduced probability of a disease. A LR− less than 0.1 provides strong evidence to rule out a diagnosis.^63^

**Figure 1:**
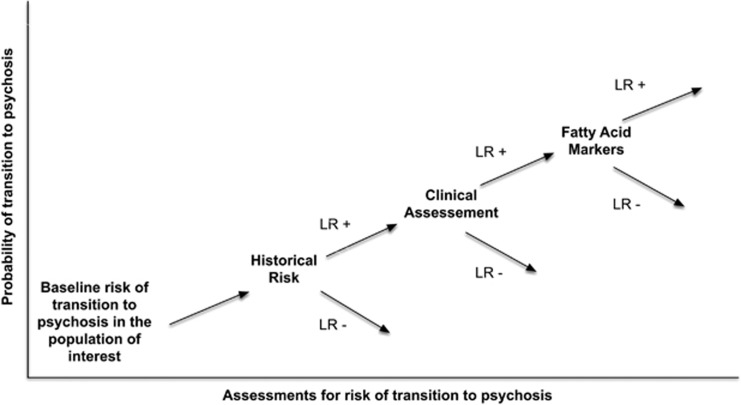
Stepwise evolution of probability of transition with new information using the odds ratio form of Bayes Rule model. LR+, positive likelihood ratio; LR−, negative likelihood ratio.

As a third step, variables were combined into logical assessment groups based on the type of data: history of drug use (historical); PANSS-positive, -negative and general scores (clinical); GAF score (clinical); total omega-3 (alpha-linolenic acid, eicosapentaenoic acid, docosapentaenoic acid and docosahexaenoic acid); and nervonic fatty acids (biomarkers). Starting with the baseline odds of transition in the population of interest, LRs (positive or negative depending on the result) were combined sequentially to determine the post-test odds for each case. The post-test odds were then transformed to post-test probability. One case that had not transitioned at 12 months was excluded from the analysis because of missing data. Figure 1 provides a graphical representation of the evolution of transition risk across modes of assessment using the model. Each new finding either increases (LR+) or decreases (LR−) the probability of transition. The steps in the calculation of the odds ratio model used the following formulae: (1) pretest odds=probability of transition /(1−probability of transition); (2) odds of transition=pretest odds × LR history of drug use × LR clinical assessment (LR PANSS-positive × LR PANSS-negative × LR PANSS general × LR GAF) × LR fatty-acid markers (LR nervonic acid × LR total omega-3 fatty acids); (3) probability of transition=odds of transition/ (1+odds of transition). ROCs were calculated for each assessment group and for factorial combinations of these groups again using the Youden Index to identify the optimal model threshold.^59^ Pairwise comparisons between these curves were calculated using the method of Delong *et al.*^58^

To visualize the utility of adding each subsequent mode of testing and explore misclassified cases, we plotted evolving probability of transition for each case.^64, 65^ In these probability plots, the assessment group (historical, clinical and biomarker) was represented on the *x* axis, and the probability of transition from UHR to psychosis on the *y* axis (Figure 1).

## Results

### Sample characteristics

Transition rate to psychosis assessed 12 months after entry into the trial was 28% (*n*=11) in the analyzed UHR cohort (*n*=40). Eight patients transitioned to schizophrenia, paranoid type; 1 to schizophreniform disorder, 1 to schizoaffective and 1 to bipolar I disorder with psychotic features. Age range was 12.9–22.3 years. The sample was predominantly early youth-aged females with high rates of smoking and regular alcohol use. One-third was treated with antidepressant medication. Sixty-two percent had a family history of psychiatric disorder, with nearly one-third a family history of depression and 15% a family history of psychosis. The predominant presenting UHR symptoms were attenuated psychotic symptoms (55%), with 33% reporting both attenuated symptoms and transient psychosis (see Supplementary Table 1).

### ROC analysis implicates a limited number of historical, clinical and biological variables as predictors of transition to psychosis

Drug use was the only significant historical predictor of transition indicated by ROC analysis (Table 1). PANSS-positive, -negative and general psychosis symptom scores, and the GAF scores were significant clinical predictors. Of the blood biomarkers, only total omega-3 and nervonic acid levels significantly predicted transition to psychosis. Variance was similar in transitioned and non-transitioned groups for all continuous variables included in the final model. Differences were larger for PANSS-negative, -positive and total omega-3 (see Supplementary Table 2). No individual oxidative markers or qEEG parameters were associated with transition. qEEG was available for 34 subjects, and this group included nine individuals who transitioned to psychosis.

### Odds ratio modeling suggests that the combination of historical, clinical and biological variables can improve the accuracy of prediction of transition to psychosis

To develop the odds ratio model, we then calculated the sensitivity, specificity and LRs for variables that significantly predicted transition to psychosis (Table 2). Individually, each predictor's LR+ and LR− was small. General PANSS score was the strongest positive predictor at 3.01, whereas high nervonic acid was the strongest negative predictor of transition at 0.17. The specificity of individual markers was low, ranging from 51 to 72%. In contrast, sensitivity was relatively high, ranging from 72 to 90%.

Using the odds ratio form of Bayes' Rule we combined significant predictors of transition into relevant groups, and then explored the accuracy of each group and of all possible group combinations using ROCs (see Table 3). All models were significant predictors of transition. Clinical assessment using the PANSS and GAF was the most accurate individual predictor group based on AUROC, followed by the fatty-acid biomarkers and history of substance use. Combining modes of data improved model performance in all cases. Pairwise comparisons of AUROC for each of these models indicated that all permutations of combined assessment modes were significantly superior to fatty acid or historical data alone (see Supplementary Table 3).

The most accurate and specific model, including history, clinical assessment and fatty-acid markers, reached a sensitivity of 72.73 at a specificity of 96.43. The bootstrapped 95% confidence interval for model sensitivity with a fixed specificity of 96.43 equaled 36.36–90.91, suggesting that the model is internally valid. At this sensitivity with a sample transition rate of 28%, the positive predictive value equaled 88.5% and negative predictive value equaled 90.3%. Suggesting both positive and negative test results would be accurate in ~90% of cases. When historical, clinical and fatty-acid markers were combined, the total model LR+ was strong at 17.82, whereas the LR− was moderate at 0.38.

### Probability plots support a stepped approach to assessment of transition risk in clinical practice

**Figure 2:**
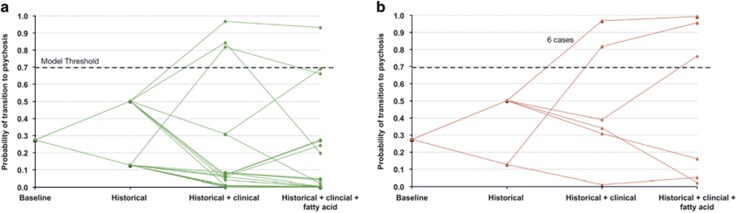
Plots of probability of transition to first episode of psychosis (FEP), given historical, clinical and biomarker information. (**a**) Stepwise probability of transition for individual cases not transitioned to psychosis at 1 year. (**b**) Stepwise probability of transition for individual cases transitioned to psychosis at 1 year.

Figure 2 shows probability plots for each patient of the analyzed cohort, calculated with the full odds ratio model (history+clinical+fatty acid) in a stepwise manner from the baseline probability of transition of 28%. Figure 2a illustrates that at a threshold probability of 0.6894 the model produced only 1 false-positive (above model threshold) in cases that did not transition. Figure 2b indicates three false-negative cases below model threshold. When the three groups of predictors were combined, the majority of cases were identified correctly as low risk (24/28 at probability <0.1) or high risk (8/11 at probability >0.75). Fatty-acid biomarkers improved accuracy in some cases at an intermediate probability of transition based on clinical data. In general, where conditional probability following historical and clinical assessment was very low (<10%) or very high (>90%), biomarker results had little impact on final probability, suggesting that fatty acids have low utility in these groups.

## Discussion

Our study demonstrates that a probabilistic model combining historical and clinical data with blood fatty-acid levels could be used to improve the specificity of UHR criteria and reduce the associated false-negative rate. A recent meta-analysis showed that the specificity of current UHR criteria lies between 59 and 67%.^66^ In this sample only 28% of those identified as UHR transitioned in 1 year. In comparison, our model identified 72.73% of these transitions at a high specificity of 96.43%, resulting in only one false-positive prediction. In translation, lowering of the false-positive rate for FEP prediction could improve the effectiveness and minimize the risks of indicated prevention with known interventions.^67^ Risk enrichment using our model also may help to reduce heterogeneity in future studies of the UHR phenotype.^68^

Our data suggest that UHR patients presenting with a high level of subthreshold psychotic symptoms and functional difficulty may represent a subgroup at higher 1-year risk for developing psychosis. In comparison, the broader group identified by current UHR criteria includes more distal risk factors such as retrospective psychotic symptom assessment and family history of psychosis.^18, 69^ On the basis of meta-analysis, Fusar-Poli *et al.*^54^ have suggested that the state-trait risk UHR criteria are not predictive of transition across studies. In our analysis of variable groups, structured clinical assessments with PANSS and GAF at baseline alone showed relatively high predictive utility (sensitivity 63.64% and specificity 92.86% Table 3). In comparison, nervonic acid and total omega-3, when combined at threshold levels identified by ROC analysis, were more sensitive (81.82%) but less specific (78.57% Table 3). Our final multimodal model, combining clinical, historical and biological variables, further improved specificity (96.43%) but not sensitivity (72.73%) as compared with the biomarker assessments alone (Table 3). These analyses allow for comparison of assessment modalities with other published approaches in improving risk prediction in UHR. For example, a 15-analyte panel for transition prediction developed from 185 candidate blood biomarkers achieved sensitivity of 60% and specificity of 90% with AUROC 0.88.^18^

Oxidative stress markers were not associated with transition. However, GSH approached significance at *P*=0.0522, suggesting that an association may be present in a larger sample or with a longer follow-up where more prodromal subjects are likely to have transitioned. Individual parameters of qEEG were also not predictive of transition in ROC analysis. Lavoie *et al.*^15^ have previously shown a significant positive correlation between negative symptoms and increased frontal delta activity in UHR patients who transitioned to psychosis in this data set. Consistent with this finding, the combination of negative symptoms and frontal delta using our model produced a sensitivity of 88.89% and specificity of 76.00% (AUROC of 0.842; *P<*0.0001), with a LR+=3.70 and LR –=0.15. However, the addition of frontal delta to the full multimodal model produced a small but nonsignificant increase in AUROC (0.929) and sensitivity (77.78%) at a specificity of 96%. These results suggest that a subset of UHR patients with negative symptoms and associated frontal slow wave pathology are at higher risk for transition. The relationship between frontal delta and transition reached significance in a larger study (*n*=113; 19.5% transition),^49^ suggesting that our analysis was underpowered.

Overall, results presented here are consistent with our previous simulation, suggesting that at least two modalities of investigation additional to UHR criteria are required to accurately differentiate the risk of transition to psychosis 1 year after presentation to a specialized clinic.^3^ Probability plots of individual cases show that the addition of fatty-acid markers was most useful where historical and clinical assessments yielded an intermediate probability of transition. A staged approach to risk assessment would then be the most efficient, using fatty-acid markers only when the probability following history and clinical assessment is between 0.1 and 0.9, that is, 23% of participants in the current study. Extending this staged approach, resource-intensive neuroimaging or electrophysiology could be reserved for cases that remain at intermediate risk based on clinical and blood biomarker assessments. In this way our modeling approximates the standard clinical assessment sequence in which investigations are ordered based on hypotheses derived from the initial bedside interview. In practice, once LRs are established and the local base rate of transition is known, our model requires only simple bedside calculations, making it suitable for implementation into the early stages of frontline clinical assessment. Further study is required to define the utility of fatty-acid levels for the prediction of omega-3 fatty-acid treatment responsiveness.^28, 31, 35, 37^ Our findings emphasize the importance of interpreting these biomarkers in the context of the clinical presentation of the patient, as should occur in effective clinical practice.^26^

### Limitations

Some caution in the interpretation of this study is warranted because of some differences in variance for predictor variables between the transition and non-transitioned groups. Ultimately, these findings need validation in a larger, longitudinal, prospective clinical sample. Particularly, the relationships of increased frontal slow waves and of GSH level to FEP need further exploration in a larger data set with complete information. Further longitudinal assessment of the single false-positive case could help to clarify whether the follow-up period was too short to identify transition. It is possible that FEP occurred shortly after the 12-month review. We were able to explore this case visually using the probability plot (see Figure 2a) and found a high risk of transition based on history and clinical assessment, with low nervonic acid but above threshold omega-3 levels. The three false-negative cases were at low to intermediate probability of transition based on clinical assessment, and two of the three cases had low risk based on fatty-acid markers. Given the heterogeneity in psychotic illness, the underlying mechanisms of psychosis in this group may not be related to fatty-acid metabolism and 100% accuracy may be difficult to achieve with many investigations in those with low-risk clinical presentation. Exploration of such subgroups may help to uncover new etiological mechanisms and identify specific markers that could be combined in sequence with fatty acids to improve prediction accuracy. Our model's focus on transition excludes other important primary outcomes such as psychosocial functioning, which may be impaired at long-term follow-up regardless of transition status.^70^ Poor functioning at first presentation as indicated by the GAF score was a predictor of transition to psychosis in our sample. Early interventions to improve psychosocial function are an important part of the management of those at high risk. Our simple Bayesian approach could also be applied to the prediction of functional outcomes to facilitate indicated functional prevention strategies.

## Conclusions

This explorative analysis suggests that the specificity and false-negative rate of predicting transition from UHR to FEP can be improved by combining historical and clinical data with fatty-acid levels using a simple probabilistic model. In translation, a reduction in false-negative rate would lead to more certainty around the implementation of indicated prevention and to risk enrichment in future UHR studies. The priming of the model with historical and clinical data was important to optimize specificity and overall accuracy. Fatty-acid biomarkers had limited value when risk of transition was very high or very low based on history and structured clinical assessment using the PANSS and GAF. If our model was replicated in independent UHR samples, a possible implication for an individual patient could be that a staged assessment protocol using a combination of bedside clinical assessment followed by fatty-acid markers is likely to be the most efficient approach to separate patients at either high or low risk of a first psychotic episode over the coming 12 months. Whereas those patients at an estimated higher risk should receive more assertive intervention with evidence-based treatments, the group at low risk may not require an assertive approach.

## Figures and Tables

**Table 1:** Receiver operating characteristic curve statistics and cutoff thresholds for transition predictors

*Variable group*	*Variables*	n	*Youden index*	*Score threshold*	*AUROC*	P
Historical	Gender	40	0.05329	1	0.527	0.7613
	Age	40	0.2414	⩽17.2841	0.517	0.8563
	Family history of psychosis	39	0.06207	1	0.531	0.6758
	Duration of symptoms at presentation	40	0.2571	⩽7	0.57	0.5125
	**Any history of drug use**^a^	**40**	**0.4514**	**1**	**0.726**	**0.006**^b^
Clinical assessment	**PANSS positive**	**40**	**0.4734**	**>14**	**0.699**	**0.0226**^b^
	**PANSS negative**	**40**	**0.4169**	**>12**	**0.723**	**0.027**^b^
	**PANSS general**	**40**	**0.4859**	**>31**	**0.768**	**0.0043**^b^
	MADRS	40	0.3448	>12	0.666	0.0664
	**GAF—low**	**40**	**0.4263**	⩽**60**	**0.774**	**0.0002**^b^
Blood biomarkers	**Nervonic acid**	**39**	**0.5357**	⩽**0.2902**	**0.688**	**0.0235**^b^
	PLA2	39	0.276	⩽0.6473	0.562	0.6056
	**Total Omega 3**	**39**	**0.4968**	⩽**5.0727**	**0.724**	**0.0405**^b^
	SOD	36	0.24	>0.0318	0.502	0.9859
	GSH	36	0.3855	⩽40.024	0.691	0.0522
	GSSG	36	0.1455	⩽16.25	0.527	0.7958
	GSH/GSSG	36	0.2036	⩽1.877	0.571	0.5142
qEEG measures	Delta frontal	34	0.2933	⩽1.82	0.542	0.7482
	Alpha frontal	34	0.1733	⩽1.71	0.507	0.9573
	Beta1 occipitoparietal	34	0.2800	⩽1.6	0.636	0.2111

Abbreviations: AUROC, area under the receiver operating characteristic curve; GAF, Global assessment of functioning; GSH, glutathione; GSSG, glutathione disulfide (oxidized form); MADRS, Montgomery Asberg Depression Rating Scale; PANSS, Positive And Negative Symptom Scale; PLA2, Phospholipase A2; qEEG, resting quantitative electroencephalography; SOD, superoxide dismutatse.

For continuous variables cutoff thresholds were set at the optimum balance between sensitivity and specificity as determined by calculation of the Youden index. The threshold value for dichotomous variables was 1.

a Any history of drug use=illicit, tobacco or alcohol.

b Significant predictors of transition at *P*<0.05 are in bold.

**Table 2:** Sensitivity, specificity and likelihood ratios used in the odds ratio form of Bayes' Rule model

*Variable group*	*Variables*	*Sensitivity (%)*	*Specificity (%)*	*LR+*	*LR−*
Historical	Any history of drug use	72.73	72.41	2.64	0.38
Clinical	PANSS positive	81.82	65.52	2.37	0.28
	PANSS negative	72.73	68.97	2.34	0.40
	PANSS general	72.73	75.86	3.01	0.36
	GAF—low	90.91	51.72	1.88	0.18
Fatty acid marker	Nervonic acid	90.91	53.57	1.96	0.17
	Total Omega-3	81.82	67.86	2.55	0.27

Abbreviations: GAF, Global assessment of function; LR−, negative likelihood ratio; LR+, positive likelihood ratio; PANSS, Positive And Negative Symptom Scale.

**Table 3:** Receiver operating characteristic curve statistics for prediction of transition to psychosis

*Model variable groups*	*Threshold probability*^a^	*AUROC*	*Sensitivity (%)*	*Specificity (%)*	*s.e.*	*95% CI AUROC*	*P AUROC*
Any history of drug use	>0.126	0.721	72.73	72.41	0.0828	0.554–0.852	0.0076
Any history of drug use+fatty-acid markers	>0.0709	0.86	90.91	75	0.067	0.712–0.950	<0.0001
Fatty-acid markers	>0.1672	0.8	81.82	78.57	0.0775	0.642–0.911	0.0001
Any history of drug use+clinical	>0.0847	0.891	90.91	82.14	0.0593	0.750–0.968	<0.0001
Clinical	>0.6705	0.864	63.64	92.86	0.0607	0.716–0.952	<0.0001
Clinical+fatty-acid markers	>0.4933	0.898	72.73	92.86	0.0577	0.758–0.971	<0.0001
Any history of drug use+clinical+fatty-acid markers	>0.6894	0.919	72.73	96.43	0.0469	0.786–0.982	<0.0001

Abbreviations: AUROC, area under the receiver operating characteristic curve; CI, confidence interval; Clinical markers, Positive and Negative Symptom Scale Positive, Negative and General scales and Global Assessment of Functioning; fatty acid, total omega 3 and nervonic acid; Historical, history of any drug use.

a Threshold probability calculated using the Youden Index.
