# Supplementary material for: Prediction of transition from ultra-high risk to first-episode psychosis using a probabilistic model combining history, clinical assessment and fatty-acid biomarkers
Source: Transl Psychiatry. 2016 Sep 20;6(9):e897–. doi: 10.1038/tp.2016.170 (PMC5048208; doi:10.1038/tp.2016.170)
Supplement: Supplementary Table 1 [file tp2016170x1.doc]

| **Group** | **Characteristic** | **Descriptor (n, %)** |
| --- | --- | --- |
| Demographics | Age (mean, SD) | 16 (1.7) |
|  | Male (n, %) | 13 (33) |
| Substance use | Tobacco use (n, %) | 24 (60) |
|  | Alcohol use <= weekly (n, %) | 23 (58) |
|  | Cannabis use (n, %) | 6 (5) |
| Medication use | Antidepressant (n, %) | 13 (33) |
|  | Benzodiazepine/sedative (n, %) | 3 (8) |
| Clinical high risk criteria | Attenuated psychotic symptoms (n, %) | 22 (55) |
|  | Transient psychosis (n, %) | 3 (8) |
|  | Trait plus state risk factors | 0 |
|  | Attenuated plus transient (n, %) | 13 (33) |
|  | Attenuated plus trait plus state (n, %) | 2 (5) |
| Family history of psychiatric disorder | Psychosis | 6 (15) |
|  | Non psychotic bipolar | 0 |
|  | Non psychotic depression | 12 (31) |
|  | Other | 6 (16) |
| Baseline symptoms | PANSS positive (mean, SD) | 14.2 (3.1) |
|  | PANSS negative (mean, SD) | 13.6 (6.5) |
|  | PANSS global (mean, SD) | 29.4 (6.6) |
|  | PANSS total (mean, SD) | 57.2 (13.9) |
|  | MADRS (mean, SD) | 18.8 (8.7) |
|  | GAF (mean, SD) | 60 (13.1) |

**Supplementary Table 1: Baseline characteristics for the UHR sample.**

Abbreviations: PANSS – positive and negative symptoms scale; MADRS - Montgomery–Åsberg Depression Rating Scale; GAF – Global Assessment of Function Scale
