# Supplementary material for: Prediction of transition from ultra-high risk to first-episode psychosis using a probabilistic model combining history, clinical assessment and fatty-acid biomarkers
Source: Transl Psychiatry. 2016 Sep 20;6(9):e897–. doi: 10.1038/tp.2016.170 (PMC5048208; doi:10.1038/tp.2016.170)
Supplement: Supplementary Table 2 [file tp2016170x2.doc]

| **Predictor** | **12-Month Transition Status** | | | | | | | | | | | | | | | |
| --- | --- | --- | --- | --- | --- | --- | --- | --- | --- | --- | --- | --- | --- | --- | --- | --- |
|  | **No Transition** | | | | | | | | **Transition** | | | | | | | |
|  | **N** | **Mean** | **95% CI** | **Variance** | **SD** | **Median** | **Minimum** | **Maximum** | **N** | **Mean** | **95% CI** | **Variance** | **SD** | **Median** | **Minimum** | **Maximum** |
| PANSS General Scale | 28 | 27.321 | 25.221 to 29.422 | 29.3373 | 5.4164 | 28.000 | 18.000 | 37.000 | 11 | 34.091 | 29.500 to 38.681 | 46.6909 | 6.8331 | 33.000 | 21.000 | 43.000 |
| PANSS Negative Scale | 28 | 11.964 | 9.892 to 14.036 | 28.5542 | 5.3436 | 11.000 | 7.000 | 32.000 | 11 | 17.273 | 12.016 to 22.529 | 61.2182 | 7.8242 | 17.000 | 7.000 | 33.000 |
| PANSS Positive Scale | 28 | 13.607 | 12.317 to 14.897 | 11.0622 | 3.3260 | 14.000 | 7.000 | 20.000 | 11 | 15.455 | 14.132 to 16.777 | 3.8727 | 1.9679 | 16.000 | 11.000 | 19.000 |
| GAF | 28 | 63.393 | 58.555 to 68.231 | 155.6548 | 12.4762 | 62.500 | 40.000 | 90.000 | 11 | 50.909 | 43.425 to 58.393 | 124.0909 | 11.1396 | 55.000 | 30.000 | 65.000 |
| Total Omega-3 | 28 | 5.481 | 5.139 to 5.822 | 0.7757 | 0.8807 | 5.251 | 3.823 | 8.123 | 11 | 4.859 | 4.064 to 5.654 | 1.4011 | 1.1837 | 4.755 | 3.438 | 7.043 |
| Nervonic Acid | 28 | 0.286 | 0.212 to 0.360 | 0.03674 | 0.1917 | 0.320 | 0.0471 | 0.737 | 11 | 0.155 | 0.0860 to 0.225 | 0.01064 | 0.1031 | 0.0859 | 0.0500 | 0.301 |

**Supplementary Table 2: Descriptive statistics for variables in the final model by transition status**

Abbreviations: PANSS – positive and negative symptoms scale; GAF – Global Assessment of Function Scale; 95% CI – confidence interval, SD – standard deviation
