# Supplementary material for: Prediction of transition from ultra-high risk to first-episode psychosis using a probabilistic model combining history, clinical assessment and fatty-acid biomarkers
Source: Transl Psychiatry. 2016 Sep 20;6(9):e897–. doi: 10.1038/tp.2016.170 (PMC5048208; doi:10.1038/tp.2016.170)
Supplement: Supplementary Table 3 [file tp2016170x3.doc]

| **Significant pairwise comparisons of AUROC^i^** | **Difference between areas** | **SE** | **95% CI** | **p** |
| --- | --- | --- | --- | --- |
| (Historical + clinical) - Historical alone | 0.17 | 0.0721 | 0.0291 to 0.312 | p = 0.0181 |
| (Historical + clinical + fatty acid) - Historical alone | 0.198 | 0.091 | 0.0196 to 0.376 | p = 0.0296 |
| (Clinical + fatty acid) - Fatty acid alone | 0.0974 | 0.0434 | 0.0124 to 0.182 | p = 0.0247 |
| (Historical + fatty acid) - Fatty acid alone | 0.06 | 0.0298 | 0.00162 to 0.119 | p = 0.0440 |
| (Historical + clinical + fatty acid) - Fatty acid alone | 0.119 | 0.0536 | 0.0134 to 0.224 | p = 0.0271 |

**Supplementary Table 3: Significant pairwise comparisons of Area Under the Receiver Operating Characteristic Curves for prediction of transition to psychosis**

^I^ Using the method of Delong et al. ^53^
